# Associated Factors for Non-Diagnostic Cytopathology in the Endobronchial Ultrasound-Transbronchial Needle Aspiration: A Retrospective Cohort Study

**DOI:** 10.3390/diagnostics16101509

**Published:** 2026-05-15

**Authors:** Umran Ozden Sertcelik, Ebru Sengul Parlak, Habibe Hezer, Eren Goktug Ceylan, Ahmet Sertcelik, Ayşegul Karalezli

**Affiliations:** 1 Department of Chest Diseases, Faculty of Medicine, Ankara Yıldırım Beyazıt University, Ankara 06800, Türkiye; aysegulkaralezli@aybu.edu.tr; 2 Department of Chest Diseases, Ankara Bilkent City Hospital, Ankara 06800, Türkiye; ebrusengul.serefparlak@sbu.edu.tr (E.S.P.); hoflaz@yahoo.com (H.H.); eren807ceylan@gmail.com (E.G.C.); 3 Department of Chest Diseases, Gülhane Faculty of Medicine, Health Sciences University, Ankara 06010, Türkiye; 4 Department of Infectious Diseases and Clinical Microbiology, Division of Epidemiology, Faculty of Medicine, Ankara Yıldırım Beyazıt University, Ankara 06800, Türkiye; ahmetsertcelik@aybu.edu.tr; 5 Department of Infectious Diseases and Clinical Microbiology, Ankara Bilkent City Hospital, Ankara 06800, Türkiye

**Keywords:** endoscopic ultrasound-guided fine-needle aspiration, delayed diagnosis, mediastinal lymph node

## Abstract

**Introduction:** Endobronchial ultrasound-guided transbronchial needle aspiration (EBUS-TBNA) is widely used for diagnosing pulmonary diseases causing mediastinal lymphadenopathy. However, non-diagnostic results may occur. This study investigated factors associated with non-diagnostic cytological results in EBUS-TBNA. **Methods:** This retrospective study included patients who underwent EBUS-TBNA at a tertiary hospital between March 2019 and December 2023. Data on demographics, biopsy techniques, cyto-/histopathological results, sonographic lymph node measurements, and pre-procedural PET-CT SUVmax values were recorded. Cytological results were classified as diagnostic or non-diagnostic. We analyzed the characteristics and associated factors of patients who were non-diagnostically identified. **Results:** Among 776 patients undergoing EBUS-TBNA, 502 (64.7%) were male, with a mean age of 61.5 ± 12.6 years. A total of 1110 lymph nodes were sampled. Of the patients, 14.1% had a non-diagnostic cytology. Among the diagnosed patients, cytological findings showed 58.9% non-malignant, 41.1% malignant. The most sampled station was station 7 (72.9%), with an average of 5.9 ± 1.4 aspirations. Diagnostic cases had significantly more aspirations (*p* = 0.022) and sampled larger lymph node sizes (*p* < 0.001). Each 1 mm increase in lymph node size raised the likelihood of diagnostic results by 1.04 times (adjOR = 1.04, 95% CI = 1.02–1.08, *p* = 0.002). The largest lymph node size significantly predicted diagnostic results (AUROC = 0.611, *p* < 0.001). A cut-off of 19.55 mm had 67.0% sensitivity and 52.2% specificity. **Conclusions:** Sampled larger lymph nodes increase diagnostic yield in EBUS-TBNA, reducing the need for repeat procedures and enabling earlier treatment, thereby decreasing morbidity and mortality.

## 1. Introduction

The widespread use of thoracic-computed tomography (CT) for the evaluation of pulmonary pathologies has led to the increasingly frequent identification of mediastinal lymph nodes of pathological size, often as incidental findings. This is particularly relevant for individuals with a history of smoking or a family history of malignancy. Once infectious aetiologies have been excluded, endobronchial ultrasound-guided transbronchial needle aspiration (EBUS-TBNA) is commonly performed to evaluate potential malignancy in patients presenting with mediastinal lymphadenopathy (LAP) [1]. Additionally, EBUS-TBNA has become an essential tool for staging lung cancer. Over time, its use has expanded beyond malignancy diagnosis, including applications in lymphoma, granulomatous diseases, infectious pulmonary disorders, and occupational lung diseases [2,3].

Although EBUS-TBNA is superior to conventional transbronchial needle aspiration (TBNA) for diagnosing intrathoracic lymphadenopathy, it may sometimes yield non-specific pathological findings, making it difficult to distinguish between malignant and benign lesions [4,5]. In such cases, further invasive investigations are often necessary, prolonging the diagnostic process.

The EBUS-TBNA procedure typically involves multiple aspirations, with a recommendation to perform at least four aspirations from each mediastinal lymph node station [6]. Nevertheless, in some cases, tissue samples may be insufficient for each aspiration, raising concerns regarding the adequacy of the samples for diagnosis. In such cases, additional aspirations may be attempted to obtain sufficient material; however, cytological results may remain non-diagnostic despite these efforts [7,8].

This study aims to investigate the factors associated with non-diagnostic cytological results in patients who underwent EBUS-TBNA. Identifying such factors may allow clinicians to anticipate non-diagnostic outcomes, expedite subsequent diagnostic procedures, and ultimately facilitate earlier diagnosis and timely initiation of appropriate treatment.

## 2. Materials and Methods

### 2.1. Patients

This study was a retrospective cohort study. Patients aged 18 years and older who underwent EBUS between 14 March 2019, and 31 December 2023, at the Chest Diseases Department of Ankara Bilkent City Hospital were included in the study. Ankara Bilkent City Hospital is a tertiary referral center with a total capacity of 4050 beds and 958 intensive care unit (ICU) beds. The hospital has a Chest Disease inpatient service with 70 beds and 23 ICU beds [9]. Approximately two to three procedures are performed daily in the bronchoscopy unit during weekdays. All patients who underwent EBUS-TBNA during the specified period were eligible for inclusion. Patients with missing cytology data were planned to exclude. Cytological results were retrieved from the hospital’s electronic record system.

### 2.2. Variables

Demographic data, including age and sex, as well as details regarding the biopsy procedures performed (forceps biopsy, bronchial lavage, bronchial brushing, transthoracic fine needle aspiration biopsy) were recorded for the patients who underwent EBUS. Histopathological and cytological results of the procedures were also noted. The number of EBUS-TBNA stations, the ultrasonographic millimetric measurements of the lymph nodes at each station, the number of aspirations performed per sampled station, and the total number of stations from which biopsies were taken were obtained from the EBUS reports.

Cytological results were classified as non-diagnostic and diagnostic. Diagnostic cytological findings were further grouped as malignant (primary—metastasis), and non-malignant (granuloma, anthracosis, and non-specific). Non-diagnostic results were defined those yielding inadequate cytological material or containing only bronchial epithelial cells, mucus or blood cells [10]. The total number of biopsied stations (total biopsied stations) and the total number of samples obtained (total biopsy count) were calculated. The total number of adjacent biopsy techniques (forceps biopsy, bronchoalveolar lavage, brush biopsy, transthoracic needle biopsy) used in conjunction with EBUS-TBNA for cytology was also recorded (total adjacent biopsy technique). If the patient had undergone a pre-procedural 18F-fluorodeoxyglucose positron emission tomography-computed tomography (18F-FDG PET-CT) within the last month, the maximum standardized uptake value (SUV-max) of the largest sampled mediastinal lymph node was recorded.

### 2.3. Endobronchial Ultrasound-Transbronchial Needle Aspiration Procedure

Real-time EBUS-TBNA was performed in all included patients using a convex probe EBUS bronchoscope (model-EU-ME2 7602092; Olympus Ltd., Tokyo, Japan). The procedures were carried out by three interventional pulmonologists (U.O.S., E.S.P., H.H.) with a minimum of four years of experience in EBUS-TBNA.

During pre-procedural preparation for EBUS, patients with co-morbidities were assessed in consultation with an anesthesiologist and relevant specialists. All patients were instructed to discontinue medications that may increase bleeding before the procedure, and platelet counts and international normalized ratio (INR) levels were assessed. Solid food intake was restricted for at least six hours before the procedure and fluid intake was stopped four hours before the procedure. Routine intravenous access was provided to all patients before the intervention and oxygen support was maintained throughout the procedure. All procedures were performed under continuous hemodynamic monitoring and deep sedation on a day-surgery basis in the bronchoscopy unit with operating theater facilities. A mouthpiece was applied to all patients prior to the procedure to facilitate bronchoscope insertion and airway protection. Following the procedure, all patients were observed for a minimum of two hours under the supervision of the anesthesiologist until the effects of sedation had fully resolved, after which discharge was performed. Laryngeal mask airway (LMA) insertion and endotracheal intubation are not routinely performed at our center; furthermore, neither was required under emergency conditions during any of the procedures in this study.

Target lymph nodes were punctured using a dedicated 22-gauge TBNA needle (NA-201SX-4022; Olympus Ltd.). The number of stations sampled and the number of aspirates per node were determined at the operator’s discretion. During each procedure, all mediastinal stations were evaluated; the long axis of each lymph node was measured and recorded perpendicular to the short axis. Aspiration sampling was performed from the appropriate stations based on clinical judgment and the suspected diagnosis.

### 2.4. Statistical Analysis

Categorical variables were presented as numbers and percentages. Quantitative variables were tested for normality using visual methods (histograms, Q-Q plots) and statistical tests (Shapiro–Wilk test). Due to the non-normal distribution of the data, descriptive statistics were reported using the median and interquartile range (IQR) for quantitative variables. Pairwise comparisons were made using Pearson’s Chi-Squared test or Fisher’s exact test for categorical variables, and the Mann–Whitney U test for quantitative variables. A binary logistic regression model was built to determine factors associated with non-diagnostic cytology in EBUS-TBNA. The goodness of fit was assessed using the Hosmer–Lemeshow test. Adjusted odds ratios (aORs) and 95% confidence intervals (CIs) were reported.

In multivariate analysis, the diagnostic performance of the maximum biopsied lymph node size for predicting diagnostic cytological outcomes was evaluated using receiver operating characteristic (ROC) analysis. The optimal cut-off point was determined as the point where the Youden index was maximized, and sensitivity, specificity, positive predictive value (PPV), negative predictive value (NPV), and accuracy were calculated for this cut-off value.

A *p*-value of less than 0.05 (two-sided) was considered statistically significant. Data imputation was not performed for missing data, and statistical analysis was completed with valid data. Data were analyzed using the Statistical Product and Service Solutions (SPSS) version 23.0 (IBM Corp., Armonk, NY, USA).

A post hoc power analysis was performed for the Wilcoxon–Mann–Whitney test (two groups) with G*power version 3.1 (Heinrich Heine Universität Düsseldorf, Düsseldorf, Germany).

## 3. Results

**Figure 1:**
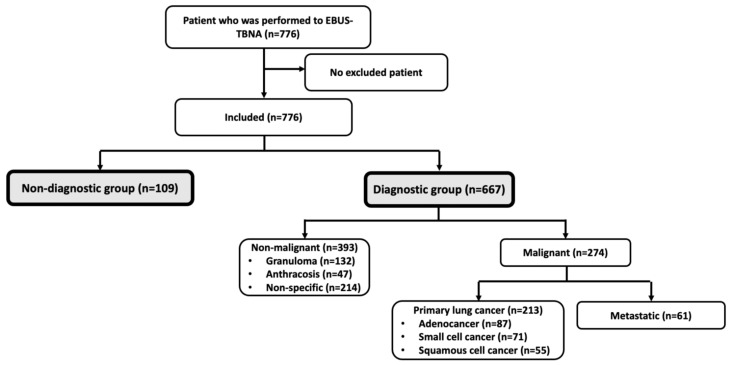
Flow chart of the study.

Between 14 March 2019, and 31 December 2023, a total of 776 patients underwent EBUS-TBNA procedures at the Department of Chest Diseases of Ankara Bilkent City Hospital. There were no patients who met the exclusion criteria, and the analyses were performed on all eligible patients (n = 776) (Figure 1).

### 3.1. Patient Characteristics

Among the patients who underwent EBUS-TBNA, 502 (64.7%) were male, and the mean age was 61.5 ± 12.6 years. Bronchial lavage was performed in 633 (81.6%) patients, with 560 (88.5%) having non-malignant cytology. Forceps biopsy was performed in 147 (18.9%) patients, with 67 (46.9%) showing non-malignant results. Bronchial brushing was performed in 85 (11%) patients, of whom 38 (44.7%) had non-malignant results. Transthoracic needle aspiration biopsy was performed in 47 (6.1%) patients, with 16 (34.7%) showing non-malignant cytology. A total of 1110 lymph nodes were sampled and evaluated cytologically. Among the EBUS-TBNA patients, sampling was performed from station 4R in 192 (24.7%) cases and from station 7 in 566 (72.9%) cases. Cytological results from the EBUS-TBNA procedure showed that 667 (86.0%) were diagnostic, and 109 (14%) were non-diagnostic. Of the diagnostic group, 393 (58.9%) were non-malignant, 274 (41.1%) were malignant. Patient characteristics and procedural details are summarized in Table 1 and Figure 1.

Regarding the size of lymph nodes at the measured stations, the average size of the lymph node at station 4R was 13.8 ± 8.3 mm, at station 7 it was 18.4 ± 10.6 mm, and at station 10R it was 13.6 ± 7.9 mm. In 564 patients who underwent EBUS-TBNA from station 7, the mean number of samples taken was 5.9 ± 1.4. Among 494 patients who underwent PET-CT, the median (IQR) FDG SUV max value was 9.5 (9.8). Distributions of age, lymph node size (mm) at each station sampled, total number of stations sampled, number of additional cyto-/histopathological diagnostic procedures, and FDG SUV max values are summarized in Table 2.

Among the 776 patients who underwent EBUS-TBNA, 109 (14.1%) had non-diagnostic cytology results, while 667 (85.9%) had diagnostic cytology results. Of the 109 non-diagnostic patients, 91 underwent additional diagnostic procedures including BAL cytology (n = 89), brush biopsy (n = 13), forceps biopsy (n = 16), and transthoracic needle aspiration biopsy (n = 8) based on clinical and radiological assessment; the remaining 18 patients were followed up with chest CT. No patient underwent repeat EBUS-TBNA or mediastinoscopy during the study period. When comparing the non-diagnostic and diagnostic groups, the median age was 65.0 (11.5) years in the non-diagnostic group, and 63.0 (16.0) years in the diagnostic group, with a statistically significant difference between the two groups (*p* = 0.018). In the diagnostic group, the number of aspirates from stations 4R, 10R, and total aspiration count were significantly higher compared to the non-diagnostic group (*p* = 0.017, *p* = 0.041, *p* = 0.022, respectively). Furthermore, the size of the lymph nodes in stations 10R, 11R, and 7, as well as the size of the maximum aspirated lymph node, were significantly larger in the diagnostic group compared to the non-diagnostic group (*p* = 0.003, *p* = 0.034, *p* = 0.001, *p* < 0.001, respectively). Distributions of age, gender, additional procedures, EBUS-TBNA station site, lymph node size (mm), aspiration number, total station number, and 18 F-PET-CT FDG SUV-max values for the non-diagnostic and diagnostic groups are presented in Table 3. In the power analysis for maximum biopsied lymph node size, the power of the study was 97%.

### 3.2. Factors Associated with Non-Diagnostic Cytology Results in EBUS-TBNA Patients

Binary logistic regression analysis was performed to determine factors associated with non-diagnostic cytology results in EBUS-TBNA patients. After controlling for age, male gender, total biopsy count, and the maximum biopsied lymph node size, each 1 mm increase in lymph node size was associated with a 1.04-fold increase in the likelihood of obtaining a diagnostic cytology result (adjusted OR = 1.04, 95% CI = 1.02–1.08, *p* = 0.002). The results of univariate and multivariate analyses are presented in Table 4.

### 3.3. Performance of Lymph Node Size as a Diagnostic Factor

**Figure 2:**
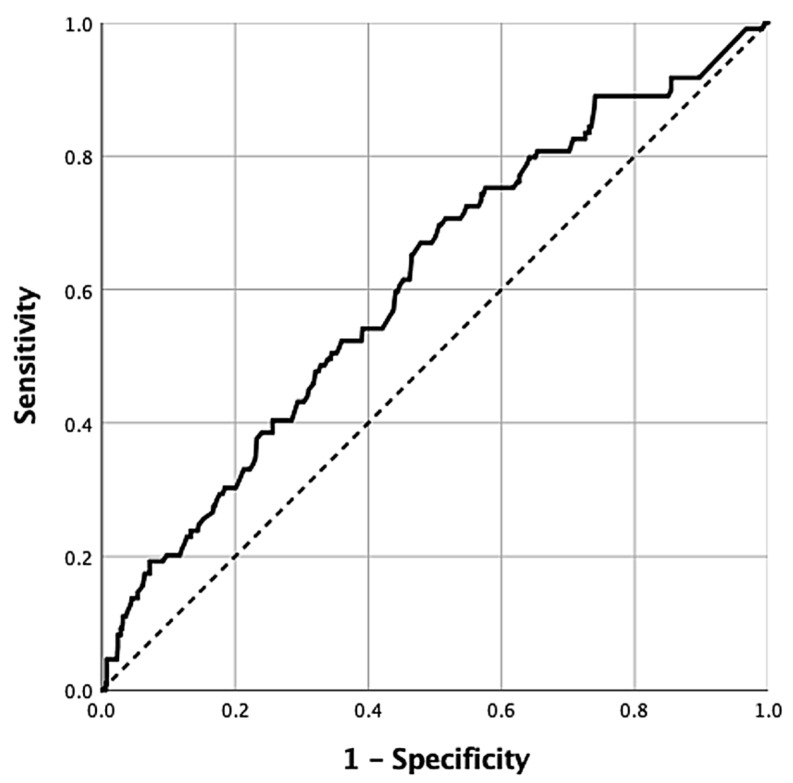
Receiver operating characteristic (ROC) curve for prediction of diagnostic cytology by maximum lymph node size sampled by endobronchial ultrasound-transbronchial needle aspiration (EBUS-TBNA).

In multivariate analysis, the diagnostic performance of the largest biopsied lymph node size for predicting a diagnostic cytological outcome was assessed using receiver operating characteristic (ROC) analysis. The area under the ROC curve (AUROC) was found to be 0.611 (95% CI = 0.554–0.668, *p* < 0.001) (Figure 2). At the optimal cut-off point of 19.55 mm, the diagnostic sensitivity was 67.0%, specificity was 52.2%, positive predictive value (PPV) was 90.6%, negative predictive value (NPV) was 18.6%, and accuracy was 54.3%.

## 4. Discussion

This retrospective cohort study evaluated factors associated with non-diagnostic cytological results of biopsy samples obtained through EBUS-TBNA at a reference healthcare center. The principal finding was that non-diagnostic outcomes were significantly associated with the maximum sampled lymph node. Each 1 mm increase in the maximum sampled lymph node size was associated with a 1.04-fold increase in the probability of a diagnostic result. Furthermore, a maximum lymph node size below 19.55 mm was correlated with non-diagnostic results, yielding a sensitivity of 67.0% and specificity of 52.2%. The relatively modest AUROC of 0.611 and the low negative predictive value (18.6%) at the 19.55 mm cut-off indicate that lymph node size alone has limited discriminative ability as a standalone predictor of diagnostic yield in EBUS-TBNA. The low specificity (52.2%) further reflects the considerable overlap in lymph node sizes between diagnostic and non-diagnostic cases, suggesting that size-based triage alone is insufficient in clinical practice.

A retrospective, single-center study conducted in 2023 included 505 patients undergoing EBUS-TBNA for the first time [6]. Only patients who had at least two samples taken from each station were included in the analysis. The biopsy samples were classified into two groups: those yielding tissue cores and those without. Cytological diagnoses were categorized as malignancy, granuloma, and non-specific patterns. In their study, Sun et al. found that 214 (42.4%) patients were diagnosed with neoplasms, 158 (31.3%) had granulomas, and 133 (26.3%) had non-diagnostic results (6). Similarly, in the present study, 274 (41.1%) patients received a malignancy diagnosis. The lower rate of non-diagnostic results in our cohort may reflect the older mean age of our patients and a higher likelihood of more advanced disease at presentation compared with the study of Sun et al. [6]. The malignant BAL cytology rate of 26.6% among patients with a final malignancy diagnosis reflects our tertiary referral center’s patient profile, where centrally located tumors and endobronchial involvement are more prevalent, increasing the yield of BAL cytology for malignant cells.

In the present study, the total number of biopsies was statistically significantly higher in the diagnostic group. A retrospective Japanese study of 275 patients undergoing radial probe EBUS-TBNA demonstrated lesion size, sheath size, ROSE (Rapid On-Site Evaluation) availability, fluoroscopic visibility, and total sample count as contributors to diagnostic accuracy. Differences in device type (radial vs. convex probe), fluoroscopy use, and ROSE availability may account for variations between that study and ours; nonetheless, lesion size was consistently associated with diagnostic outcomes, in keeping with our findings [11]. However, consistent with our findings, lesion size was found to be associated with the diagnostic outcomes.

The present study also found that the number of EBUS-TBNA aspirations was higher in the diagnostic group. Lee et al. investigated the minimum number of aspirations required to achieve optimal diagnostic performance [8]. In their study of 102 patients with chronic obstructive pulmonary disease (COPD), 162 lymph nodes with a short diameter greater than 5 mm were sampled, and four aspirations per case were performed. The diagnostic accuracy rates for the first to fourth aspirations were 89.7%, 94.4%, 98.4%, and 98.4%, respectively. Prospective studies have similarly demonstrated that increasing the number of aspirations improves diagnostic yield, with four aspirations generally proving sufficient [8]. In a study conducted in Korea (2019–2022) involving 228 lung cancer patients, factors affecting the diagnostic yield of EBUS-TBNA were investigated. The results found that an increased number of aspirated samples (*p* < 0.001) and a larger lymph node diameter (*p* = 0.016) were significantly associated with successful tissue obtainment [12]. In the present study, however, since over 95% of patients received four or more biopsies, the marginal benefit of additional passes may have been insufficient to demonstrate a statistically significant association between biopsy count and diagnostic success in multivariate analysis. Likewise, sex, which was significant on univariate analysis, did not retain significance in the multivariate model.

In the present study, 41% of patients ultimately diagnosed with malignancy had diagnostic EBUS-TBNA results, highlighting the contribution of lymph node size to diagnostic success. The median maximum lymph node size was 24.7 mm (IQR 13.2) in the malignant group and 17.7 mm (IQR 12.1) in the non-malignant group. Nevertheless, two-way ANOVA revealed no statistically significant interaction between malignancy status and diagnostic outcome (*p* = 0.625); consequently, results from both groups were analyzed together.

A study from India sampled mediastinal-hilar lymph nodes with a SUVmax of 2.5 or greater in 57 patients with extrathoracic malignancies and correlated cytological findings with lymph node size [13]. The mean size of abnormal lymph nodes (metastatic + granulomatous) was 17.5 (minimum–maximum = 8–35) mm, and the mean SUVmax was 9.1 (3.4–18), and these findings were statistically significant when compared with reactive (normal) lymph nodes. At a size threshold of 13 mm, the sensitivity, specificity, positive predictive value (PPV), and negative predictive value (NPV) for detecting abnormal lymph nodes were 75.5%, 65%, 75%, and 72%, respectively [13]. Another study examining various sonographic features in predicting metastasis found that at a cut-off of 10 mm, the sensitivity was around 25% and the specificity was around 95% [14]. At a cut-off point of 14 mm, sensitivity and specificity were above 60% and diagnostic accuracy was 82.2% [14]. In this study, sensitivity for obtaining a diagnostic cytological result was 67.0%, specificity was 52.2%, PPV was 90.6%, NPV was 18.6% and accuracy was 54.3% for lymph node sizes greater than 19.55 mm. Consistent with the literature, the median SUVmax value of sampled lymph nodes in the diagnostic group was found to be 9.8 (IQR = 9.7).

A systematic review examining the relationship between diagnostic delay and prognosis in primary lung cancer found that delays are associated with more advanced disease stages, postponed surgery, and increased mortality [15]. In this context, the ability to anticipate non-diagnostic EBUS-TBNA outcomes is of considerable clinical importance, as it may enable earlier adoption of alternative diagnostic strategies, particularly in patients with malignancy, metastasis, lymphoma, or sarcoidosis, thereby facilitating timely treatment and potentially reducing morbidity and mortality.

### 4.1. Strengths of the Study

The principal strengths of this study are its large sample size—comprising 776 patients and 1110 lymph nodes—and its investigation of factors associated with non-diagnostic cytological results in EBUS-TBNA, a topic for which no directly comparable studies exist in the literature, to the best of our knowledge. The inclusion of potential confounding factors in the multivariable model further strengthens the validity of the findings. The results may assist clinicians in identifying patients at higher risk of non-diagnostic outcomes, enabling the earlier utilization of supplementary diagnostic modalities in such cases.

### 4.2. Limitations

First, ROSE (Rapid On-Site Evaluation), which may help ensure sample adequacy and reduce unnecessary examinations, was not employed in this study [16,17]. Second, the single-center design may limit the generalizability of the findings, although it enhances internal validity. Third, retrospective collection of data from medical records introduces the potential for missing data and misclassification bias. On the other hand, no critical data were missing. The use of electronic records mitigates recall bias. The relatively short and defined data collection period also reduces the likelihood of classification inconsistencies over time. Finally, operator experience and pathologist expertise—factors known to influence EBUS-TBNA diagnostic yield—were not systematically evaluated and should be addressed in future studies.

## 5. Conclusions

While larger lymph node size is associated with higher diagnostic yield, the 19.55 mm cut-off alone has limited predictive accuracy. In patients with lymph node sizes below this threshold, the addition of complementary imaging or invasive techniques should be considered. Future multicenter studies incorporating multiparametric models—combining size, sonographic features, and metabolic activity—are needed to improve the prediction of non-diagnostic outcomes in EBUS-TBNA.

## Figures and Tables

**Table 1:** Distribution of gender, adjacent procedures to endobronchial ultrasound (EBUS), and EBUS-transbronchial needle aspiration station.

		n	%
Male gender		502	64.7
BAL		663	81.6
BAL cytology			
	Malignant	73	11.5
	No malignant	560	88.5
Forceps biopsy		147	18.9
	Malignant	76	53.1
	No malignant	67	46.9
	Non-diagnostic	4	-
Brush biopsy		85	11.0
	Malignant	47	55.3
	No malignant	38	44.7
Transthoracic needle aspiration biopsy		47	6.1
	Malignant	31	66.0
	No malignant	16	34.7
EBUS-TBNA stations			
	2R	6	0.8
	4R	192	24.7
	10R	81	10.4
	11R	94	12.1
	7	566	72.9
	4L	26	3.4
	10L	79	10.2
	11L	66	8.5
	2R	6	0.8

BAL: Bronchoalveolar lavage, EBUS-TBNA: Endobronchial ultrasound-transbronchial needle aspiration.

**Table 2:** Distribution of age, maximum lymph node sizes, total number of sampled stations, and positron emission tomography-computed tomography findings.

		n	Mean ± Std. Dev.	Median (IQR)
Age (years)		776	61.5 ± 12.6	63.0 (15.0)
Maximum size of lymph node (mm) with EBUS		776	20.7 ± 8.4	19.5 (13.8)
	4R	641	13.8 ± 8.3	11.3 (10.5)
	10R	518	13.6 ± 7.9	11.5 (10.7)
	11R	393	13.3 ± 7.4	12.0 (9.3)
	7	723	18.4 ± 10.6	16.7 (13.6)
	4L	395	9.2 ± 5.9	7.5 (5.9)
	10L	363	11.8 ± 7.3	9.4 (8.7)
	11L	300	11.6 ± 6.3	9.8 (6.4)
EBUS-TBNA sample count		774	8.1 ± 13.1	7.0 (4.0)
	4R	192	5.2 ± 1.6	5.0 (2.0)
	10R	81	5.4 ± 1.5	5.0 (2.0)
	11R	94	5.4 ± 1.7	5.0 (2.0)
	7	564	5.9 ± 1.4	6.0 (2.0)
	4L	26	5.1 ± 1.4	5.0 (2.0)
	10L	79	5.3 ± 1.6	5.0 (2.0)
	11L	66	5.7 ± 1.4	6.0 (2.0)
Total biopsied station with EBUS-TBNA		776	1.4 ± 0.6	1.0 (1.0)
Total adjacent biopsy technique		776	1.2 ± 0.8	1.0 (1.0)
18 F-PET-CT FDG SUV-max		494	13.5 ± 50.1	9.5 (9.8)

Std. dev.: standard deviation, IQR: interquartile range, EBUS-TBNA: Endobronchial ultrasound-transbronchial needle aspiration, 18 F-PET-CT FDG SUV-max: fludeoxyglucose-18 positron emission tomography-computed tomography fludeoxyglucose maximum standardized uptake value.

**Table 3:** Distribution of patient characteristics based on non-diagnostic Endobronchial ultrasound-transbronchial needle aspiration results.

		**Total** **(n = 776)**	**Non-Diagnostic (n = 109)**	**Diagnostic (n = 667)**		
		**n (%)**	**n (%)**	**n (%)**	**OR (95% CI)**	***p*** **-Value**
Male gender		502 (64.7)	76 (69.7)	426 (63.9)	1.30 (0.84–2.02)	0.236
BAL		633 (81.6)	89 (81.7)	544 (81.6)	1.01 (0.60–1.70)	0.982
Forceps biopsy		147 (18.9)	16 (14.7)	131 (19.6)	0.70 (0.40–1.24)	0.220
Brush biopsy		85 (11.0)	13 (11.9)	72 (10.8)	1.12 (0.60–2.10)	0.726
Transthoracic needle asp.		47 (6.1)	8 (7.3)	39 (5.8)	1.28 (0.58–2.81)	0.545
EBUS-TBNA stations						
	4R	192 (24.7)	25 (22.9)	167 (25.0)	0.90 (0.55–1.44)	0.637
	10R	81 (10.4)	11 (10.1)	70 (10.5)	0.95 (0.49–1.87)	0.898
	11R	94 (12.1)	16 (14.7)	78 (11.7)	1.30 (0.73–2.32)	0.376
	7	566 (72.9)	83 (76.1)	483 (72.4)	1.22 (0.76–1.95)	0.416
	4L	26 (3.4)	3 (2.8)	23 (3.4)	0.80 (0.23–2.69)	>0.999
	10L	79 (10.2)	8 (7.3)	71 (10.6)	0.67 (0.31–1.42)	0.290
	11L	66 (8.5)	6 (5.5)	60 (9.0)	0.59 (0.25–1.40)	0.226
	2R	6 (0.8)	1 (0.9)	5 (0.7)	1.23 (0.14–10.64)	0.598
		**Non-Diagnostic**		**Diagnostic**		
		**n**	**Median (IQR)**	**n**	**Median (IQR)**	***p*** **-Value**
Age (years)		109	65.0 (11.5)	667	63.0 (16.0)	**0.018**
EBUS-TBNA stations						
	4R mm	25	19.9 (16.3)	167	17.0 (12.9)	0.354
	4R bx count	25	4.0 (1.5)	167	5.0 (2.0)	**0.017**
	10R mm	11	11.0 (4.3)	70	19.4 (14.7)	**0.003**
	10R bx count	11	4.0 (3.0)	70	6.0 (1.3)	**0.041**
	11R mm	16	13.5 (8.6)	78	15.0 (10.7)	**0.034**
	11R bx count	16	5.0 (1.8)	78	5.5 (3.0)	0.249
	7 bx mm	83	15.3 (10.0)	483	19.4 (13.2)	**0.001**
	7 bx count	82	6.0 (1.0)	482	6.0 (2.0)	0.273
	4L mm	3	15.1 (-)	23	15.0 (17.0)	0.880
	4L bx count	3	5.0 (-)	23	5.0 (2.0)	0.762
	10L mm	8	15.6 (7.7)	71	18.1 (12.6)	0.338
	10L bx count	8	5.5 (2.8)	71	5.0 (3.0)	0.679
	11L mm	6	15.3 (11.3)	60	12.0 (9.3)	0.330
	11L bx count	6	5.0 (2.3)	60	6.0 (2.0)	0.330
	2R mm	1	-	5	11.7 (7.5)	0.667
	2R bx count	1	-	6	4.0 (2.8)	0.571
Total bx count		109	7.0 (3.0)	666	8.0 (4.0)	**0.022**
Max size of biopsied LN, mm		109	16.3 (11.2)	667	19.9 (13.9)	**<0.001**
Total biopsied station with EBUS-TBNA		109	1.0 (1.0)	667	1.0 (1.0)	0.526
Total adjacent biopsy technique		109	1.0 (0.0)	667	1.0 (1.0)	0.734
18 F-PET-CT FDG SUV-max		64	7.7 (9.8)	430	9.8 (9.7)	0.165

BAL: Bronchoalveolar lavage, asp.: aspiration, EBUS-TBNA: Endobronchial ultrasound-transbronchial needle aspiration, IQR: interquartile range, bx: biopsy, max: maximum, LN: lymph node, 18 F-PET-CT FDG SUV-max: fludeoxyglucose-18 positron emission tomography-computed tomography fludeoxyglucose maximum standardized uptake value. Statistically significant *p*-values are shown in bold.

**Table 4:** Factors associated with cytologically non-diagnostic findings in patients with endobronchial ultrasound-transbronchial needle aspiration.

	Univariate		Multivariate *	
	OR (95% CI)	*p*-Value	adjOR (95% CI)	*p*-Value
Age, year	1.02 (1.00–1.04)	**0.029**	1.02 (1.00–1.03)	0.086
Male gender	1.30 (0.84–2.02)	0.237	1.27 (0.81–1.99)	0.290
Total bx count	0.92 (0.86–0.99)	**0.023**	0.94 (0.87–1.01)	0.077
Max biopsied LAP, mm	0.95 (0.93–0.98)	**<0.001**	0.96 (0.93–0.98)	**0.002**

* Hosmer–Lemeshow test *p*-value = 0.825. Statistically significant *p*-values are shown in bold.

## Data Availability

The datasets used and/or analyzed during the current study are available from the corresponding author upon reasonable request.

## Abbreviations

aOR	Adjusted Odds Ratios
AUROC	The Area Under the ROC curve
CI	Confidence Intervals
CT	Computed Tomography
COPD	Chronic Obstructive Pulmonary Disease
EBUS-TBNA	Endobronchial Ultrasound-guided Transbronchial Needle Aspiration
ICU	Intensive Care Unit
IQR	Interquartile Range
ROC	Receiver Operating Characteristic
NPV	Negative Predictive Value
PPV	Positive Predictive Value
ROSE	Rapid On-Site Evaluation
SUVmax	Maximum Standardized Uptake Value
18F-FDG PET-CT	18F-Fluorodeoxyglucose Positron Emission Tomography-Computed Tomography
